# Preserving Cornerstones of Student's Assessment in Medical Education During COVID-19

**DOI:** 10.3389/fpsyg.2021.591152

**Published:** 2021-04-09

**Authors:** Pedro Tadao Hamamoto Filho, Angélica Maria Bicudo, Dario Cecilio-Fernandes

**Affiliations:** ^1^Department of Neurology, Psychology and Psychiatry, Universidade Estadual Paulista (UNESP), Botucatu, Brazil; ^2^Department of Pediatrics, University of Campinas (UNICAMP), Campinas, Brazil; ^3^Department of Medical Psychology and Psychiatry, University of Campinas (UNICAMP), Campinas, Brazil

**Keywords:** assessment and education, COVID-19, remote (distant) education, medical education, evaluation

Since the outbreak of the pandemic caused by COVID-19, there was a significant disruption in our day-to-day life. To control the spread of COVID-19, several measures were taken, including social distancing and isolation. All educational systems were seriously compromised, and medical education, in particular, was heavily hit by those measurements, leading to the challenge of redesigning the current form of teaching. Worldwide, there was a trend of moving all the learning and teaching activities to online. The transition from face-to-face to online activities was not an easy task, especially for low- and middle-income countries (Cecilio-Fernandes et al., 2020). Overall, the transition focuses on the knowledge part of the curriculum and a few medical skills, which were predominantly skills that required non-motor skills, such as communication and clinical reasoning. Although an essential part of the medical curriculum is work-based learning, there is still a need to assess students, as we come to the end of the semester. In this perspective paper, we highlight different methods of assessment and how we make a sound decision about students' progression.

As online teaching has focused on knowledge, it is not fair and feasible to assess students' skills that are taught at bedside, which includes most of the medical skills, attitudes, and competence. Since a proper alignment between learning objectives, strategies, and assessment are vital for any medical curriculum (Prideaux, 2003, 2007), students' assessment should be re-aligned with the new content of teaching. Since the online learning objectives are low in Miller's pyramid, it is only possible to assess students at the level of “knowing” and “knowing how” (Miller, 1990; Wass et al., 2001). Therefore, we should only use assessment tools that are aligned with those two levels.

Different assessment methods can be used to assess the “knowing” and “knowing how” level at Miller's pyramid (Miller, 1990). Although this article will not discuss the various methods, here is a shortlist that may be used in online assessment: multiple-choice questions, essays, short answer, very short answer, open-ended questions, open-book questions, case study, oral examination, and many others. Indeed, the problem is not in the lack of options but in how we can make a fair assessment while assuring that students will not cheat in an online environment.

To answer that question, first we need to understand that end of semester tests, or having two tests a semester, was already lacking. Students often cram before end of semester tests, leading to a low retention of the material. Also, students are often overwhelmed with the number of tests at the end of semester, which forces them to study for some tests while disregarding others. With the transition to online teaching and now assessment many medical educators realized that they should use other types of assessment methods, creating an opportunity to use best practices in assessment. Many curricula focus the decision of pass/fail students on a high-stakes test. High-stakes test refers to the stake of the exam, meaning that if only one test will decide whether you pass or fail, we call it a high-stakes test (Norcini and McKinley, 2007). This type of test leads to students studying for one test, which hampers learning (Wrigley, 2012). Also, this type of test is relatively applicable in face-to-face education, since all students have to take the assessment at the same time and at the same place, which, in turn, makes cheating unlikely. In an online environment, however, we cannot control the setting, opening the door for cheating. Therefore, many medical educators are looking for different strategies of assessment for an online environment.

If the idea is to keep the high-stakes test, there are two options. First, there are online proctoring and psychometric forensic programs that can help. Those programs make it possible to watch all the students via webcam and more advanced ones also track the gaze of your eyes, making sure that students are only looking at the screen (Weiner and Hurtz, 2017). Psychometric forensic refers to track all questions to verify whether there is a change on the item psychometric pattern, during and after the test (Cizek and Wollack, 2017). Those software are expensive and require specialized workers that currently are scarce in Brazil. Second, a computerized adaptive test could be used. The computerized adaptive test refers to an algorithm that will select the next questions based on students' previous performance (Cecilio-Fernandes, 2019; Collares and Cecilio-Fernandes, 2019). This makes the test unique to every student, therefore decreasing the likelihood of cheating. Also, computerized adaptive tests identify students' knowledge gaps more precisely than the traditional format. Finally, students' motivation increases, since they will answer a test that is aligned with their level of knowledge. Although there is free software for computerized adaptive tests, there is a need to have a psychometrician with experience running this type of test. These two options may be feasible for some medical schools, but the majority may not have enough expertise or funding, due to the complexity of both solutions.

From a psychometric point of view, high-stakes tests should provide evidence of validity, reliability, and standardization. Validity refers to whether the test is measuring the proposed construct. Reliability refers to the amount of error in a test and whether the scores are reproducible over time. Standardization refers to how the scores are calculated based on a large sample (American Educational Research Association, 2014). Unfortunately, high-stakes tests in medical schools do not achieve the psychometric rigor necessary to make a fair decision, leading to an unfair decision in many times.

Shifting the paradigm from high-stakes tests to several low-stakes tests seems more realistic to our reality. Actually, rather than being concerned with the pass/fail score, we now move to the original essence of assessment: did students learn? This new paradigm also brings us close to current best guides of assessment, moving toward a system of assessment (Norcini et al., 2018) or programmatic assessment (Prideaux, 2003; Schuwirth and van der Vleuten, 2011; van der Vleuten et al., 2012, p. 14). Both of these refer to using multiple methods of assessment during different points in time. Then, all these different methods and assessment are considered for the decision of the students. Considering multiple assessments has the benefit of changing students' behavior. For example, instead of studying for one test, now they have to study across the semester or year, which, in turn, is beneficial for students' learning (Cecilio-Fernandes et al., 2017). Also, incorporating multiple methods of assessment gives a clearer evaluation of students as a whole and also decreases the likelihood of cheating, since students will have many tests. It is also possible to include clinical cases discussion, allowing a discussion between students. Using this system opens the door for many possibilities while assuring the psychometric rigor of the assessment program (Bok et al., 2018).

Making a decision on multiple tests also allows to include the formative character of assessment. By formative character, we mean feedback, since studies have shown that students feel demotivated with formative assessment compared to summative assessment. Also, there is a call to reconcile summative and formative assessment, since they are connected at their inception (Lau, 2016). Feedback is one of the most powerful tools for learning (for a review, please see Hattie and Timperley, 2007). Feedback avoids learning an incorrect knowledge by guiding students to the correct knowledge. Feedback also helps learners to identify the relevant knowledge and it motivates students by rewarding correct actions or correcting incorrect actions. Changing from high-stakes tests to low-stakes tests allows medical educators to incorporate feedback in the assessment, increasing students' learning.

Most high-stakes tests are based on closed- or open-ended questions. Using a multiple method of assessments allows other assessment methods to be included. For example, medical educators can now take the advantage of quiz at the beginning of each class, which would help teachers to identify students' knowledge gaps of previous content and also take advantage of the testing effect. Testing effect refers to students who are tested to perform better in a retention test than those who re-studied the material (Roediger and Karpicke, 2006). Also, teachers could include case discussion and group work as part of the decision making. Including different methods of assessment allows for assessing students with a different lens and given feedback on different aspects of students' knowledge.

Noteworthy, online teaching and assessment have mainly focused on knowledge. We have demonstrated that it is possible to make a fair decision on the students' future. However, this may be limited to preclinical training. For the clinical years, it is possible to teach and assess to an extent. Of course, we are going through difficult times, but a discussion about clinical training is necessary. For example, we do not believe that it is possible to teach and assess clinical skills using online tools. Even clinical reasoning that is based on knowledge, the online platform, discussion, and cases are oversimplified compared to real-life scenarios. This discussion is even more important for the 6th year students who are about to graduate. Unfortunately, there is no right answer or a simple solution. Nevertheless, we need to consider whether we should approve students even cutting at least 30% of their practical training, which is perhaps the crucial part of the medical curriculum.

Shifting from face-to-face to online activities may have brought us a unique opportunity to change our knowledge assessment practice. Changing from high-stakes assessment to multiple low-stakes assessments is an advance to the assessment field, and we hope that this change will last after the pandemic.

## Author Contributions

DC-F, AB, and PH contributed to the conceptualization, writing original draft, and writing—review and editing. All authors contributed to the article and approved the submitted version.

## Conflict of Interest

The authors declare that the research was conducted in the absence of any commercial or financial relationships that could be construed as a potential conflict of interest.
